# Is the risk of second primary malignancy increased in multiple myeloma in the novel therapy era? A population-based, retrospective cohort study in Taiwan

**DOI:** 10.1038/s41598-020-71243-z

**Published:** 2020-09-01

**Authors:** Yanfang Liu, Hsin-An Hou, Hong Qiu, Chao-Hsiun Tang

**Affiliations:** 1 Global Epidemiology, Janssen Research & Development, 2 Science Park Drive, Sinagapore, 118222 Singapore; 2 Division of Hematology, Department of Internal Medicine, National Taiwan University Hospital, No 7, ZhongShan South Road, Zhongzheng District, Taipei, 100 Taiwan; 3 Global Epidemiology, Janssen Research & Development, 1125 Trenton-Harbourton Road, Titusville, NJ 08560 USA; 4 School of Health Care Administration, College of Management, Taipei Medical University, No.172-1 Keelung Road, Section 2, Taipei, 106 Taiwan

**Keywords:** Risk factors, Haematological cancer

## Abstract

Longer survival in patients with multiple myeloma (MM) after treatment with novel agents (NA) such as thalidomide, bortezomib, and lenalidomide may be associated with increased risks of developing second primary malignancies (SPM). Few data describe the risk of SPM in patients with MM in Asia. This population-based retrospective cohort study assessed the risk of SPM in MM using the Taiwan National Cancer Registry and National Health Insurance Research databases from 2000 to 2014. Among 4,327 patients with newly diagnosed MM initiated with either novel agents alone (NA), chemotherapy combined with novel agents (CCNA), or chemotherapy alone (CA), the cumulative incidence of SPM overall was 1.33% at year 3. The SPM incidence per 100 person-years (95% confidence interval [CI]) was 0.914 (0.745–1.123) overall, 0.762 (0.609–1.766) for solid tumours, and 0.149 (0.090–0.247) for haematological malignancies. We compared risks of SPM using a cause-specific Cox regression model considering death as a competing risk for developing SPM. After controlling for age, gender, Charlson Co-morbidity Index, and time-period, the risk of developing any SPM or any haematological malignancy was significantly reduced in patients initiated on NA (2010–2014 period) compared to chemotherapy alone (adjusted hazard ratio 0.24, 95% CI 0.07–0.85, and 0.10, 95% CI 0.02–0.62, respectively). Contemporary treatment regiments using NA (mainly bortezomib) were associated with a lower risk for a SPM in comparison with CA.

## Introduction

Multiple myeloma (MM) is clonal plasma cell malignancy that is heterogenous in its clinical presentation and progression. It is more common in older age-groups and in men. Survival following MM has improved since the availability of novel immunomodulatory drugs including thalidomide and lenalidomide, and proteasome inhibitors such as bortezomib, combined with increasing use of high-dose therapy prior to autologous stem cell transplantation (ASCT)^1,2^. Longer survival may be associated with an increased risk of second primary malignancies (SPMs)^3^. Patients with MM are at higher risk of developing SPMs compared to the risk of cancer in the general population, possibly contributed to by the older age of these patients, the predominance of men, genetic susceptibility, other environmental or behavioural factors and exposure to MM treatment^4,5^. Exposure to alkylating agents such as melphalan or cyclophosphamide increases the risk of developing acute myeloid leukaemia (AML) and myelodysplastic syndrome (MDS)^3^. A report by the Medical Research Council in the United Kingdom in 1987 identified a 3% risk of developing a hematological malignancy for each year of melphalan therapy^6^. Combined treatment with lenalidomide and melphalan increases the risk of a SPM more than melphalan alone, with a cumulative incidence of 6.9% at 5 years^7^. The development of a SPM is associated with shorter survival than without SPM. A population-based study in Sweden that assessed patients over a 50-year period estimated a 2.3-fold higher risk of death in patients with MM who developed a SPM, and a 4.9-fold risk if the second tumour was a haematological malignancy^8^. Survival in patients with MM who developed AML or MDS was lower than in patients who developed the malignancy de novo.

Taiwan has a centralized health insurance system that reimburses healthcare for all residents. Using claims data from the National Health Insurance Research database (NHIRD) we showed that the unadjusted incidence of MM in Taiwan increased by 30% from 2007 to 2012, accompanied by a decrease in case fatality from 25.5 to 19.4% that coincided with the availability of novel agents for MM treatment in Taiwan^9^. There are concerns that the incidence of SPM will increase as survival from MM improves.

In this retrospective cohort study, we conducted an in-depth study of SPMs in patients with MM, using the Taiwan Cancer Registry, covering the time period before and after the introduction of novel agents for first-line treatment of MM in Taiwan. Uniquely, we linked cancer registry data with patients’ treatment data from NHIRD claims data to evaluate the risk of SPM in patients who received different MM treatments for first-line therapy.

## Methods

### Data source

The Taiwan Cancer Registry was founded in 1979 and is organized and funded by the Ministry of Health^10^. The registry is operated by the National Public Health Association, which maintains an expert advisory board to standardize definitions, coding, and procedures within the reporting system. All hospitals in Taiwan with at least 50 beds mandatorily report all newly diagnosed and confirmed malignancies to the registry^11^. Highly detailed information on provision of care, treatment and outcomes is collected from 80 hospitals covering more than 90% of all cancer cases diagnosed annually in Taiwan. Data in the registry are linked to death certificates, the National Health Insurance Catastrophic Illness Registry and cancer screening programs to identify missing cancer cases. Diagnoses are coded in the International Classification of Diseases for Oncology, 3rd Edition (ICD-O-3) format and data are validated through rigorous internal quality control^11^.

Data from the Taiwan Cancer Registry were electronically linked to the NHIRD, which holds all data from the National Health Insurance system, which covers almost the entire population and records claims data on all medical services provided in Taiwan^12^. Primary and secondary diagnoses are coded using International Classification of Diseases, Ninth Revision, Clinical Modification (ICD-9-CM) format, and demographic information, date and type of service provided (physician services, drugs, prescriptions, laboratory and imaging examinations, and hospital ward) are recorded. Data quality is ensured by the National Health Insurance Administration^12–14^.

Claims data from the NHIRD were used to identify specific treatment for MM using chemotherapy, novel therapy (bortezomib, thalidomide and lenalidomide) and steroids prescribed during outpatient visits or hospital admissions. Drugs were captured by Anatomical Therapeutic Chemical codes. Death and date of death were identified in the linked Death Registry.

De-identified aggregated patient data were used for the analysis. The study was granted an exemption from ethical review by the Taipei Medical University-Joint Institutional Review Board, and an exemption from the need for patient consent. The study was conducted according to all applicable guidelines and regulations.

### Study population

All patients with a new diagnosis of MM in the cancer registry (ICD-O-3 codes M-97323 and C42) from 01 January 2000 until 30 June 2014 were included in the cohort and followed up until 31 Dec 2014. Patients were excluded if they had a record of any primary cancer prior to the MM diagnosis date or if they had a death record before the MM diagnosis date. Patients were also excluded if they had any diagnosis of plasma cell leukaemia (203.1×) or other immunoproliferative neoplasms (203.8×) within 2 months of the first diagnosis of MM due to disease progression, if treatment was not initiated by 31 December 2014, or if initial treatment was with steroids alone. Patients were initially stratified into 3 cohorts according to the first-line treatment they received; novel agents alone, chemotherapy combined with novel agents, or chemotherapy alone. Patients were followed up from the initial treatment date for the occurrence of SPMs in the Cancer Registry.

The study population was divided into three time-periods reflecting different phases in the advancement of MM treatment available in the formulary list of the NHI (Table 1). The period 2000–2004 (pre-novel agent period) was characterised the exclusive use of chemotherapeutic agents for MM treatment. Novel agents started to be used during the 2005–2009 transition period: thalidomide was reimbursed for first-line therapy of MM in July 2009 and bortezomib was approved for third-line therapy in 2007. The majority of patients in 2005–2009 continued to be initiated on first-line chemotherapy but may have received novel agents, mainly thalidomide, in second or third-line. The novel agent period from 2010–2014 was characterised by the availability of bortezomib which was reimbursed for first-line treatment of patients < 65 years of age or eligible for ASCT in 2011 and was reimbursed without restriction in 2012. Lenalidomide was reimbursed for second-line treatment in January 2012.

**Table 1:** Number and percentage of first-line treatments received by patients with multiple myeloma, by treatment period.

	Treatment period 2000–2004	Treatment period 2005–2009			Treatment period 2010–2014		
	Chemotherapy alone (n = 1,035)	Novel agent alone (n = 69)	Novel + chemo (n = 134)	Chemotherapy alone (n = 1,186)	Novel agent alone (n = 957)	Novel + chemo (n = 814)	Chemotherapy alone (n = 132)
Thalidomide + bortezomib	NA	^a^	NA	NA	365 (38.1)	NA	NA
Thalidomide-based	NA	65 (94.2)	NA	NA	473 (49.4)	NA	NA
Bortezomib-based	NA	3 (4.3)	NA	NA	119 (12.4)	NA	NA
Thalidomide + bortezomib + other chemo	NA	NA	0	NA	NA	39 (4.8)	NA
Thalidomide + other chemo	NA	NA	8 (6.0)	NA	NA	94 (11.5)	NA
Bortezomib + other chemo	NA	NA	0	NA	NA	43 (5.3)	NA
Melphalan + thalidomide + bortezomib	NA	NA	0	NA	NA	41 (5.0)	NA
Melphalan + thalidomide	NA	NA	123 (91.8)	NA	NA	536 (65.8)	NA
Melphalan + bortezomib	NA	NA	3 (2.2)	NA	NA	61 (7.5)	NA
Melphalan-based	716 (69.2)	NA	NA	813 (68.5)	NA	NA	83 (62.9)
Cyclophosphamide-based	71 (6.9)	NA	NA	82 (6.9)	NA	NA	19 (14.4)
Vincristine	50 (4.8)	NA	NA	68 (5.7)	NA	NA	10 (7.6)
Other mono chemo	^a^	NA	NA	7 (0.6)	NA	NA	^a^
Two-chemo combination	186 (18.0)	NA	NA	198 (16.7)	NA	NA	19 (14.4)
Three-chemo combination	11 (1.1)	NA	NA	18 (1.5)	NA	NA	0

Chemo refers to bendamustine, cisplatin, cyclophosphamide, doxorubicin, etoposide, vincristine.

NA, not applicable.

^a^To protect patient privacy, all non-zero counts that were less than three were suppressed.

### Outcome measures and statistical analysis

The primary study outcome was the occurrence of a SPM after initiating treatment for MM. SPM were defined as new malignant tumours diagnosed more than 180 days after the MM diagnosis date to reduce the risk of including pre-existing undiagnosed malignancies in the analysis. The incidence rate of a SPM per 100 person-years was calculated with 95% confidence interval (CI) using a competing risk model where death without prior SPM was considered the competing risk. We initially determined cause-specific hazard ratios (HR) of SPM between different treatment regimens after adjusting for age, gender, Charlson Co-morbidity Index (CCI) score and year of diagnosis (Table 2). However, we found that the interaction terms of treatment regimen and time period had significant effects on SPM, particularly haematological SPM (Table S1). Therefore, we re-grouped patients into 5 groups, dividing the novel agents alone and chemotherapy combined with novel agents treatment groups into two treatment periods: 2005–2009 and 2010–2014. Cause-specific HR of SPM between different treatment regimens were re-assessed after adjusting for age, gender, CCI score and time-period, using the chemotherapy alone cohort as the reference group.

**Table 2:** Demographic and clinical characteristics of patients with multiple myeloma by treatment regimen.

Variable	All patients	Novel agent alone	Novel + Chemo	Chemotherapy alone	*p*
	(N = 4,327)	(N = 1,026)	(N = 948)	(N = 2,353)	
**Demographic characteristics**					
Gender n (%)					0.07
Male	2,503 (57.8)	562 (54.8)	564 (59.5)	1,377 (58.5)	
Female	1824 (42.2)	464 (45.2)	384 (40.5)	976 (41.5)	
Age mean [SD]	66.3 [11.8]	64.4 [12.2]	69.6 [10.8]	65.9 [11.8]	< 0.001
< 65	1828 (42.2)	557 (54.3)	291 (30.7)	980 (41.6)	
≥ 65	2,499 (57.8)	469 (45.7)	657 (69.3)	1,373 (58.4)	
**Clinical characteristics n (%)**					
Year of diagnosis					< 0.001
2000	172 (4)	^b^	0 (0)	171 (7.3)	
2001	216 (5)	0 (0)	0 (0)	216 (9.2)	
2002	223 (5.2)	0 (0)	0 (0)	223 (9.5)	
2003	237 (5.5)	0 (0)	0 (0)	237 (10.1)	
2004	230 (5.3)	0 (0)	0 (0)	230 (9.8)	
2005	252 (5.8)	4 (0.4)	0 (0)	248 (10.5)	
2006	246 (5.7)	3 (0.3)	3 (0.3)	240 (10.2)	
2007	302 (7)	7 (0.7)	7 (0.7)	288 (12.2)	
2008	274 (6.3)	13 (1.3)	15 (1.6)	246 (10.5)	
2009	344 (8)	71 (6.9)	136 (14.3)	137 (5.8)	
2010	377 (8.7)	139 (13.5)	197 (20.8)	41 (1.7)	
2011	409 (9.5)	161 (15.7)	213 (22.5)	35 (1.5)	
2012	421 (9.7)	250 (24.4)	153 (16.1)	18 (0.8)	
2013	426 (9.8)	251 (24.5)	157 (16.6)	18 (0.8)	
2014^a^	198 (4.6)	126 (12.3)	67 (7.1)	5 (0.2)	
**Comorbidities associated with MM n (%)**					
Renal impairment	606 (14)	186 (18.1)	148 (15.6)	272 (11.6)	< 0.001
Anaemia	1,314 (30.4)	327 (31.9)	283 (29.9)	704 (29.9)	0.49
Bone fracture	714 (16.5)	185 (18)	162 (17.1)	367 (15.6)	0.18
Pneumonia	507 (11.7)	136 (13.3)	118 (12.4)	253 (10.8)	0.08
**CCI Deyo mean [SD]**	1.23 [1.42]	1.29 [1.48]	1.28 [1.41]	1.19 [1.40]	0.02
CCI = 0	1784 (41.2)	420 (40.9)	353 (37.2)	1,011 (43)	
CCI = 1	1,061 (24.5)	231 (22.5)	267 (28.2)	563 (23.9)	
CCI = 2	726 (16.8)	187 (18.2)	159 (16.8)	380 (16.1)	
CCI≧3	756 (17.5)	188 (18.3)	169 (17.8)	399 (17)	

CCI, Charlson co-morbidity index; MM, multiple myeloma; N, number of patients; n (%), number (percentage) of patients with the indicated characteristic; SD, standard deviation; SPM, second primary malignancy. Novel agents refer to thalidomide and bortezomib.

^a^January 2014 to June 2014.

^b^To protect patient privacy, all non-zero counts that were less than three were suppressed.

Baseline demographic and disease characteristics were summarized using descriptive statistics. Frequencies and percentages were reported for categorical variables and the mean with standard deviation (SD) for continuous variables. An intention-to-treat analysis was performed for patients according to initial treatment cohort. The cumulative incidence for each treatment cohort was calculated using the cause-specific Cox model where death without prior SPM was considered the competing risk. All analyses were performed using SAS Version 9.4 (Cary, NC, USA).

## Results

### Characteristics of patients with MM

**Figure 1:**
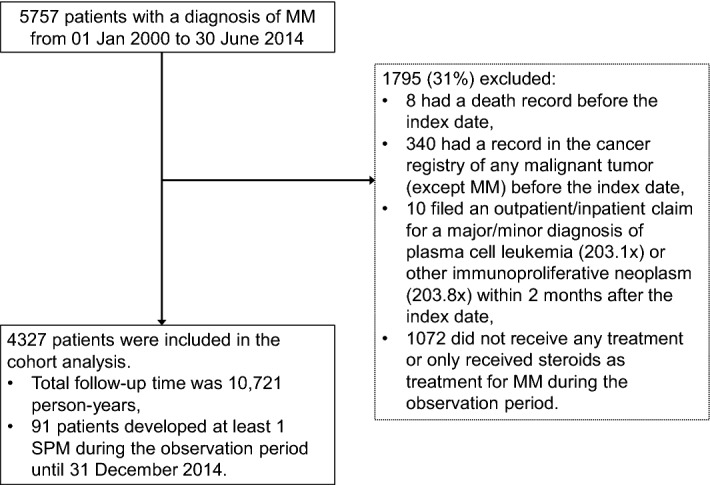
Patient selection flowchart.

There were 5,757 patients in the National Cancer Registry with a new diagnosis of MM (ICD-O-3 codes M-97323 and C42) from 01 Jan 2000 to 30 June 2014, of whom 340 were excluded because of a pre-existing malignancy prior to the MM diagnosis (Fig. 1). A total of 4,327 eligible patients were included in the cohort analysis. The mean age at diagnosis was 66.3 years (SD 11.8 years), 57.8% of patients were aged ≥ 65 years, and 57.8% were men (Table 2). At diagnosis, 30.4% of patients had anaemia, 16.5% had bone fracture, 14.0% had renal impairment and 11.7% had pneumonia.

A total of 23.7% of patients initiated treatment with novel agents alone, 21.9% initiated treatment with chemotherapy combined with novel agents, and 54.4% with chemotherapy alone. Patients treated with novel agents were younger and had higher rates of renal impairment at baseline than patients who received chemotherapy combined with novel agents or chemotherapy alone (p < 0.01 for both). The mean CCI score was lower in patients initiated with chemotherapy alone (p = 0.02). Significant differences between treatments in terms of index year reflect the different availability of novel agents over the study period and form the basis for the three time-periods used in the analysis.

### Incidence rates of SPM

The total follow-up time was 10,721 person-years. There were 91 patients (2.1%) who developed 93 SPMs during the follow-up period. Solid tumours were reported in 76 patients (83.5%) and haematological cancers in 15 patients (16.5%). Site-specific incidence rates per 100 person-years (95% CI) were 0.12 (0.07–0.21) for cancer of the colon, 0.10 (0.05–0.18) for liver/intrahepatic bile duct malignancies, 0.09 (0.05–0.17) for lung, bronchial/tracheal malignancies, and 0.06 (0.03–0.13) for myeloid leukaemia and other skin malignancies (Table 3).

**Table 3:** Incidence rates of second primary malignancies by ICD-9 code in 91 patients with multiple myeloma.

ICD9	Cancer type	No. of SPM events	Person-years	Incidence rate per 100 person-years (95% CI)
153	Colon	12	10,060.5	0.12 (0.07–0.21)
155	Liver, intrahepatic bile ducts	10	10,067.2	0.10 (0.05–0.18)
162	Trachea, bronchus, lung	9	10,075.7	0.09 (0.05–0.17)
205	*Myeloid leukaemia*	*6*	*10,075.8*	*0.06 (0.03–0.13)*
173	Other skin	6	10,070.9	0.06 (0.03–0.13)
188	Bladder	5	10,069.3	0.05 (0.02–0.12)
151	Stomach	4	10,075.7	0.04 (0.01–0.11)
174	Female breast	4	10,064.9	0.04 (0.01–0.11)
202	*Other lymphoid and histiocytic tissue*	*4*	*10,070.0*	*0.04 (0.01–0.11)*
141	Tongue	3	10,072.6	0.03 (0.01–0.09)
150	Oesophagus	3	10,074.6	0.03 (0.01–0.09)
154	Rectum, recto-sigmoid junction, anus	3	10,077.3	0.03 (0.01–0.09)
185	Prostate	3	10,075.9	0.03 (0.01–0.09)
208	*Unspecified leukaemia*	*3*	*10,068.0*	*0.03 (0.01–0.09)*
147	Nasopharynx	2	10,070.6	0.02 (0.00–0.08)
156	Gallbladder, extrahepatic bile ducts	2	10,077.1	0.02 (0.00–0.08)
182	Uterus	2	10,071.5	0.02 (0.00–0.08)
189	Kidney, urinary organs	2	10,074.2	0.02 (0.00–0.08)
199	No site specified	2	10,077.8	0.02 (0.00–0.08)
143	Gum	1	10,077.6	0.01 (0.00–0.07)
146	Oropharynx	1	10,077.5	0.01 (0.00–0.07)
148	Hypopharynx	1	10,077.8	0.01 (0.00–0.07)
184	Other/unspecified female genital organs	1	10,075.5	0.01 (0.00–0.07)
193	Thyroid gland	1	10,066.8	0.01 (0.00–0.07)
200	Lymphosarcoma, reticulosarcoma	1	10,078.0	0.01 (0.00–0.07)
204	*Lymphoid leukaemia*	*1*	*10,077.1*	*0.01 (0.00–0.07)*
238.7	*Neoplasm of uncertain behaviour of other lymphatic and hematopoietic tissues*	*1*	*10,077.9*	*0.01 (0.00–0.07)*

Italics indicates haematological malignancies.

CI, confidence intervals.

The histological distribution of SPM differed from the primary cancer types in 340 patients later diagnosed with MM who were excluded because of the presence of the primary malignancy prior to the MM diagnosis date (Table 4). Haematological malignancies made up 5.8% (n = 21) of cancers diagnosed in the years prior to the MM diagnosis date but comprised 16.5% (n = 15) of SPMs. The most frequent haematological cancer diagnosed in patients with MM was myeloid leukaemia, which moved from 25th place prior to MM diagnosis to fourth place afterwards. There was no clear trend in the distribution of solid tumours before and after the MM diagnosis date, with eight of the 10 most frequent solid tumours prior to MM diagnosis also ranked in the top 10 solid SPM.

**Table 4:** Incidence rates of second primary malignancies by ICD-9 code in 91 patients with multiple myeloma.

ICD9	Cancer type	No. of SPM events	Person-years	Incidence rate per 100 person-years (95% CI)
153	Colon	12	10,060.5	0.12 (0.07–0.21)
155	Liver, intrahepatic bile ducts	10	10,067.2	0.10 (0.05–0.18)
162	Trachea, bronchus, lung	9	10,075.7	0.09 (0.05–0.17)
205	*Myeloid leukaemia*	*6*	*10,075.8*	*0.06 (0.03–0.13)*
173	Other skin	6	10,070.9	0.06 (0.03–0.13)
188	Bladder	5	10,069.3	0.05 (0.02–0.12)
151	Stomach	4	10,075.7	0.04 (0.01–0.11)
174	Female breast	4	10,064.9	0.04 (0.01–0.11)
202	*Other lymphoid and histiocytic tissue*	*4*	*10,070.0*	*0.04 (0.01–0.11)*
141	Tongue	3	10,072.6	0.03 (0.01–0.09)
150	Oesophagus	3	10,074.6	0.03 (0.01–0.09)
154	Rectum, recto-sigmoid junction, anus	3	10,077.3	0.03 (0.01–0.09)
185	Prostate	3	10,075.9	0.03 (0.01–0.09)
208	*Unspecified leukaemia*	*3*	*10,068.0*	*0.03 (0.01–0.09)*
147	Nasopharynx	2	10,070.6	0.02 (0.00–0.08)
156	Gallbladder, extrahepatic bile ducts	2	10,077.1	0.02 (0.00–0.08)
182	Uterus	2	10,071.5	0.02 (0.00–0.08)
189	Kidney, urinary organs	2	10,074.2	0.02 (0.00–0.08)
199	No site specified	2	10,077.8	0.02 (0.00–0.08)
143	Gum	1	10,077.6	0.01 (0.00–0.07)
146	Oropharynx	1	10,077.5	0.01 (0.00–0.07)
148	Hypopharynx	1	10,077.8	0.01 (0.00–0.07)
184	Other/unspecified female genital organs	1	10,075.5	0.01 (0.00–0.07)
193	Thyroid gland	1	10,066.8	0.01 (0.00–0.07)
200	Lymphosarcoma, reticulosarcoma	1	10,078.0	0.01 (0.00–0.07)
204	*Lymphoid leukaemia*	*1*	*10,077.1*	*0.01 (0.00–0.07)*
238.7	*Neoplasm of uncertain behaviour of other lymphatic and hematopoietic tissues*	*1*	*10,077.9*	*0.01 (0.00–0.07)*

Italics indicates haematological malignancies.

CI, confidence intervals.

### Cumulative incidence of developing a SPM

The cumulative incidence of any SPM was 0.42% at year 1, 0.92% at year 2, and 1.33% at year 3 (Table 5). The 3-year cumulative incidence was 1.08% for developing a solid SPM, and 0.25% for a haematological malignancy (Table 5). The 3-year cumulative probability of developing any SPM was 0.59% in patients initiated with novel agents alone, 1.84% in patients initiated with chemotherapy combined with novel agents, and 1.42% in patients initiated with chemotherapy alone.

**Table 5:** Cumulative incidence of a second primary malignancy in patients with multiple myeloma by treatment regimen.

	Number of patients	Number of SPM events^a^	Cumulative incidence of SPM^b^		
			% at 1 year	% at 2 years	% at 3 years
**Any SPM**	4,327	91	0.42	0.92	1.33
Novel agent alone	1,026	8	0.19	0.41	0.59
Novel + chemotherapy	948	25	0.59	1.28	1.84
Chemotherapy alone	2,353	58	0.45	0.99	1.42
**Haematological malignancy**	4,327	15	0.05	0.17	0.25
Novel agent alone	1,026	3	0.05	0.17	0.25
Novel + chemotherapy	948	4	0.06	0.22	0.33
Chemotherapy alone	2,353	8	0.04	0.15	0.23
**Solid malignancy**	4,327	76	0.38	0.75	1.08
Novel agent alone	1,026	5	0.13	0.25	0.36
Novel + chemotherapy	948	21	0.53	1.06	1.51
Chemotherapy alone	2,353	50	0.42	0.83	1.19

SPM, second primary malignancy.

^a^Follow up from the date of treatment until event, death, or end of data, whichever came first.

^b^Kaplan–Meier method was used to estimate the cumulative probability of a second malignancy tumour.

### Incidence rate of SPM by treatment group

The incidence per 100 person-years (95% CI) of the first SPM after treatment initiation was 0.914 (0.745–1.123) overall, 0.762 (0.609–1.766) for solid tumours and 0.149 (0.090–0.247) for haematological tumours (Table 6). Only 15 patients developed haematological malignancies over the study period, rendering the estimates of incidence imprecise, with wide 95% CIs. The incidence (95% CI) of solid tumours per 100 person-years was 0.786 (0.596–1.038) in patients who received chemotherapy alone, and 0.252 (0.095–0.672) in patients who received novel agents during the 2010–2014 period.

**Table 6:** Cause-specific Cox regression model second primary malignancy in patients with multiple myeloma, considering death as a competing risk for developing a second primary malignancy.

Competing risk model	No. of SPM events	Person-years	Incidence rate per 100 person-years (95% CI)	Crude HR (95% CI)	*p*	Adjusted^a^ HR (95%CI)	*p*
**All patients with MM**	91	9,951	0.914 (0.745–1.123)				
Novel agent alone, 2005–2009	2	204	0.983 (0.246–3.929)	1.16 (0.28–4.79)	0.839	1.06 (0.25–4.48)	0.935
Novel agent alone, 2010–2014	6	1,583	0.379 (0.170–0.843)	0.53 (0.23–1.27)	0.154	0.24 (0.07–0.85)	0.027
Novel + chemotherapy, 2005–2009	4	363	1.101 (0.413–2.933)	1.32 (0.47–3.67)	0.599	1.01 (0.35–2.90)	0.984
Novel + chemotherapy, 2010–2014	21	1,458	1.441 (0.939–2.210)	1.99 (1.17–3.40)	0.012	0.81 (0.28–2.36)	0.701
Chemotherapy alone	58	6,343	0.914 (0.707–1.183)	Ref = 1		Ref = 1	
**Haematological malignancy**	15	10,057	0.149 (0.090–0.247)				
Novel agent alone, 2005–2009	1	204	0.490 (0.069–3.478)	3.11 (0.39–24.9)	0.286	2.89 (0.32–26.2)	0.346
Novel agent alone, 2010–2014	2	1,589	0.126 (0.031–0.503)	1.01 (0.21–4.90)	0.987	0.10 (0.02–0.62)	0.013
Novel + chemotherapy, 2005–2009	1	365	0.274 (0.039–1.943)	1.75 (0.22–14.0)	0.598	2.24 (0.25–20.3)	0.475
Novel + chemotherapy, 2010–2014	3	1,472	0.204 (0.066–0.632)	1.58 (0.41–6.13)	0.506	0.17 (0.03–0.85)	0.031
Chemotherapy alone	8	6,427	0.124 (0.062–0.249)	Ref = 1		Ref = 1	
**Solid malignancy**	76	9,972	0.762 (0.609–0.954)				
Novel agent alone, 2005–2009	1	204	0.491 (0.069–3.486)	0.71 (0.10–5.22)	0.741	0.65 (0.09–4.84)	0.677
Novel agent alone, 2010–2014	4	1,586	0.252 (0.095–0.672)	0.43 (0.15–1.22)	0.114	0.65 (0.07–5.83)	0.699
Novel + chemotherapy, 2005–2009	3	363	0.825 (0.266–2.559)	1.22 (0.38–3.97)	0.741	0.85 (0.25–2.83)	0.788
Novel + chemotherapy, 2010–2014	18	1,461	1.232 (0.776–1.956)	2.07 (1.16–3.71)	0.014	2.75 (0.37–20.6)	0.325
Chemotherapy alone	50	6,358	0.786 (0.596–1.038)	Ref = 1		Ref = 1	

HR, hazard ratio; Ref, reference treatment. See Table 1 for other abbreviations.

^a^Adjusted by age as a continuous variable, CCI, gender, treatment period.

### Cause-specific hazard ratio of SPMs in MM patients

After controlling for age, gender, CCI and time-period (Tables 1 and 2), the risk of developing any SPM or a haematological malignancy was significantly reduced in patients initiated on novel agents within the 2010–2014 period compared to the chemotherapy alone period (adjusted HR 0.24, 95% CI 0.07–0.85; p = 0.027, and 0.10, 95% CI 0.02–0.62; p = 0.013, respectively) (Table 6). The adjusted HR for developing solid malignancies was < 1 comparing the novel agents 2010–2014 period with the chemotherapy alone period but was not statistically significant.

The risk of developing a haematological malignancy was also significant reduced in the chemotherapy combined with novel agents 2010–2014 period compared to the chemotherapy alone period (adjusted HR 0.17, 95% CI 0.03–0.85, p = 0.031).

## Discussion

This is the first population-based, retrospective cohort study using the Taiwan National Cancer Registry to estimate the cause-specific incidence of SPM in patients with newly diagnosed MM, and to link this to the initial treatment regimen. The National Cancer Registry captures the diagnosis and the date of occurrence of all cancer diagnoses, and the NHIRD captures complete treatment information for individual patients from inpatient, outpatient, and pharmacy sources. Linking patient treatment information with registry data enabled us to follow patients in different treatment exposure cohorts to estimate the incidence of SPM for each treatment group. The NHIRD captures health-related data from almost the entire population (99.7%) of Taiwan, and all cancer cases are captured in the cancer registry database. Therefore, the major strengths of our study are the completeness of the data capture achieved by linking two comprehensive, longitudinal, population-based databases, and the availability of baseline characteristics to identify potential confounding factors. Other strengths of the study are the long observation period for SPM development, and selection of study years covering a period of fundamental change in MM treatment in Taiwan. In contrast to follow up studies of clinical trial cohorts, this study used real-world data to compare different treatments in the entire MM population for a follow up period of up to 15 years. The results can be considered representative, supported by a consistent distribution of cancer among the study population versus that reported for the general population in Taiwan^15^.

For Kaplan–Meier methods used to estimate the time to development of SPMs, patients who die before experiencing a SPM are considered as censored observations, implying that a SPM could still occur over the remaining observation period even though the patient has died. This method is therefore prone to overestimating the probability of SPM, particularly in settings where the incidence of the competing event (death) is high. In the case of MM patients, the incidence of death is typically very high (case fatality rate of 18.3% in 2015^16^), as the patient population is on average elderly (mean age of 66 years at MM diagnosis in Taiwan). We used a cause-specific Cox regression in which the occurrence of SPM was the outcome of interest and death without prior SPM was considered as the competing risk. All patients who died before developing SPM were censored and underwent no further follow-up. This model is appropriate for exploring questions of aetiology^17^, and in our study allowed evaluation of the cause-specific risk of SPM for each treatment cohort.

MM treatment evolved over the study period in reflecting reimbursement for first-line treatment of MM approved for thalidomide in 2009 and bortezomib in 2011. We divided patients into 5 treatment groups on the basis of evidence of interactions between treatment regimen and time period on SPM. Prior to 2004, all patients were treated with chemotherapy alone regardless of their clinical condition and disease severity. Thereafter, the availability of first thalidomide, and then increasingly bortezomib, allowed physicians to select the optimum treatment based on the patient’s clinical picture, potentially introducing significant confounding by indication. Because of the high risk of confounding by indication from 2005, we used patients who had received chemotherapy alone as the reference treatment regimen for evaluating the risk of SPM associated with different treatments, thereby avoiding direct comparisons between treatments potentially subject to confounding. Supporting our approach is the observation that the median follow-up period of patients treated with chemotherapy alone was not longer compared to other treatment groups (data not shown). This suggests that survival bias did not play a role in our findings. We observed that treatment with novel agents alone during 2010–2014 was associated with a significantly decreased risk of any SPM when compared to chemotherapy alone. Treatment with novel agents alone during 2010–2014 and combined chemotherapy with novel agents during 2010–2014 had a significantly lower risk of haematological SPM in comparison with chemotherapy alone. The 2010–2014 period was associated with substantial use of bortezomib and reduced use of thalidomide for first-line treatment compared with the 2005–2009 time-period in which thalidomide predominated. Thalidomide has been linked to an increased risk of SPM^18,19^, whereas the International Myeloma Working Group (IMWG) consensus of the available literature that concluded that the risk of SPM following treatment with bortezomib was low, and in some studies consistent with background rates^3^. The mixed results observed in the chemotherapy combined with novel agents treatment groups in the 2005–2009 versus the 2010–2014 periods are difficult to interpret, reflecting in varying degrees the transition from thalidomide use to bortezomib use, and different median age suggesting substantial effects of confounding by indication. For example, in 2005–2009, patients receiving chemotherapy combined with novel agents were older (mean 69.4 years) than both other treatment groups (mean 60.6 years in the novel agents alone group and 65.8 years in the chemotherapy alone group), reflecting the use of thalidomide as a chemotherapy-sparing drug for patients too old or frail to tolerate an aggressive chemotherapy regimen or ASCT. Supporting this hypothesis, the ASCT rate was 10.1% in the chemotherapy combined with novel agents versus 19.6% in the novel agents alone group. Age was a confounding factor in the analysis and was adjusted when we compare the HR among the different treatment groups.

The most frequently reported cancers prior to a MM diagnosis were consistent with those reported by Chiang et al.^15^, (albeit with some variation in the ranking), who used the Taiwan Cancer Registry to assess cancer incidences in the whole population^15^. Seven of the 10 malignancies with the highest age-standardized incidence rates were shared, with thyroid cancer, uterine cancer and oral cancers potentially under-represented and skin, cervical and bladder cancer possibly over-represented in patients with a malignancy diagnosed prior to MM. However, in comparison with the distribution of SPMs, we observed an increase in haematological cancers, especially myeloid leukaemia, in patients with MM. Myeloid leukaemia ranked as the fourth most common SPM in patients with MM. By comparison, myeloid leukaemia in the general population of Taiwan (2012) ranked 44th^15^.

Several studies have found that the incidence of SPM in patients with MM is similar to the risk of cancer in the general population, but that the site-specific incidence rates differ significantly. These include a population-based matched cohort study that also used the NHIRD^20^, and a SEER-based study in the United States^21^. In Taiwan, patients with MM had an 11.5-fold higher incidence of hematologic malignancies and a 2.1-fold lower incidence of solid tumours than patients without MM^20^. Similarly, the US SEER study found the risk of developing a solid tumour was decreased in patients with MM (standardized incidence ratio [SIR] 0.94, 95% CI 0.89–0.99), whereas the risk of a haematological malignancy was increased (1.68, 95% CI 1.46–1.92)^21^. A second SEER-based study confirmed a significantly lower risk of some solid tumours (prostate and breast) and an increase in haematological malignancies that was most significant for AML (SIR 6.51, 95% CI 5.42–7.83)^22^. The authors observed no change in the risk of solid or haematological SPM in patients with MM before or after the introduction of novel agents for MM treatment, but observed higher rates of SPM in younger patients, possibly due to the use of aggressive treatment regimens, although an 80% increased risk of AML in the first 2 years after the MM diagnosis points to other non-treatment-related contributing factors^22^. In a population-based study in Sweden, patients with MM had an increased risk of developing a SPM compared to the general population (SIR 1.26, 95% CI 1.16–1.36) which was highest for AML/MDS (SIR 11.51, 95% CI 8.19–15.74)^4^.

To protect patient privacy, all non-zero counts that were less than three were suppressed in the presentation of results. However, this had no effect on calculations of incidence or SPM risk. Other potential study limitations included a lack of clinical staging information which did not allow us insights into patterns of SPM presentation in Taiwan. We conducted an intention-to-treat analysis in which the risk of SPM was assessed based on the initial treatment received. Any potential impact of subsequent treatments that might have contributed to a SPM was not assessed. For example, lenalidomide was approved for re-imbursement of patients with treatment failure to first-line therapy in December 2012, and has been associated with an increased risk of SPM, particularly when combined with melphalan^3^.

Highlights of the IMWG into SPM in MM were that the overall risk of SPM is patients with MM is low and the pathogenesis is likely to be multifactorial^3^. The potential risk of SPM should not alter the therapeutic decision-making process. Nevertheless, the availability of novel agents appears to be favourable for the prognosis of MM in terms of SPM development, and previous estimates of SPM risk in MM are unlikely to be applicable in the new treatment environment. Our study showed that the incidence of SPM in MM is low but provides evidence of fewer SPM following first-line treatment with novel agents compared to chemotherapy or chemotherapy combined with novel agents. These data provide useful information for physicians selecting treatments that optimize long term safety of patients with MM. This study shows that linking high quality databases increases the breadth and depth of the knowledge that can be gained from analyses of real-world data and has important implications for future clinical research.

## Supplementary information

Supplementary Table S1.

## Data Availability

The data underlying this study are from the National Health Insurance Research Database, Taiwan Cancer Registry, and Death Registry, which have been transferred to the Health and Welfare Data Science Center (HWDC). The Taiwan government prohibits release of the aforementioned datasets to the public domain. Interested researchers can obtain the data through formal application to the HWDC, Department of Statistics, Ministry of Health and Welfare, Taiwan (https://dep.mohw.gov.tw/DOS/np-2497-113.html).
