# Supplementary material for: Is the risk of second primary malignancy increased in multiple myeloma in the novel therapy era? A population-based, retrospective cohort study in Taiwan
Source: Sci Rep. 2020 Sep 1;10:14393. doi: 10.1038/s41598-020-71243-z (PMC7463238; doi:10.1038/s41598-020-71243-z)
Supplement: Supplementary file 1 — Supplementary Table S1. [file 41598_2020_71243_MOESM1_ESM.pdf]

Is the risk of second primary malignancy increased in multiple myeloma in the novel therapy era? A population-based, retrospective cohort study in Taiwan.

Authors: Yanfang Liu MD, MPH, Hsin-An Hou MD, Hong Qiu MD, PhD, and Chao-Hsiun Tang, PhD

SUPPLEMENT

Supplementary Table S1. Cause-specific Cox regression model of second primary malignancy in patients with multiple myeloma, considering death as a competing risk for developing SPM- before and after interaction terms of treatment regimen and time period were included

	Crude HR (95% CI)	*p-value*	Adjusted[a] HR (95%CI)	*P-value*	Adjusted[b] HR (95%CI)	*P-value*
**Any SPM**	-	-			-	-
Novel agent alone	0.62 (0.29-1.32)	0.212	0.37 (0.14-0.98)	0.046	0.24 (0.07-0.85)	0.027
Novel+chemotherapy	1.84 (1.11-3.03)	0.017	1.08 (0.51-2.32)	0.837	0.81 (0.28-2.36)	0.701
Chemotherapy alone (reference)	Ref=1	-	Ref=1		Ref=1	-
Novel agent alone (time period 2005-2009)					4.46 (0.66-30.33)	0.13
(Novel+chemotherapy) (time period 2005-2009)					1.25 (0.28-5.58)	0.77
**Haematological malignancy**	-	-				
Novel agent alone	1.33 (0.34-5.10)	0.682	0.31 (0.05-1.88)	0.203	0.10 (0.02-0.62)	0.013
Novel+chemotherapy	1.64 (0.49-5.54)	0.425	0.45 (0.08-2.41)	0.349	0.17 (0.03-0.85)	0.031
Chemotherapy alone (reference)	Ref=1	-	Ref=1		Ref=1	-
Novel agent alone (time period 2005-2009)					28.44 (1.65-489.12)	0.02
(Novel+chemotherapy) (time period 2005-2009)					13.09 (0.86-198.91)	0.06
**Solid malignancy**	-	-			-	-
Novel agent alone	0.47 (0.18-1.20)	0.112	0.32 (0.11-0.95)	0.040	0.65 (0.07-5.83)	0.699
Novel+chemotherapy	1.88 (1.08-3.25)	0.025	1.15 (0.54-2.43)	0.723	2.75 (0.37-20.63)	0.325
Chemotherapy alone (reference)	Ref=1	-	Ref=1		Ref=1	-
Novel agent alone (time period 2005-2009)					1.01 (0.05-19.65)	1.00
(Novel+chemotherapy) (time period 2005-2009)					0.31 (0.03-3.22)	0.33

HR, hazard ratio; Ref, reference treatment.

[a]Adjusted by age as a continuous variable, CCI, gender, treatment period.

[b]Adjusted by age as a continuous variable, CCI, gender, treatment period, and interaction terms of treatment group and time period.
